# The efficacy psychobiotics for depression treatment

**DOI:** 10.1192/j.eurpsy.2023.1267

**Published:** 2023-07-19

**Authors:** Y. Denysov, G. Putyatin, S. Moroz, V. Semenikhina

**Affiliations:** 1 Psychiatry, psychotherapy, addictology and medical psychology, Donetsk national medical university, Kropyvnitskyi; 2 Psychiatry, Municapal Enterprise “Dnipro Multiprofile Clinical Hospital for the Provision of Psychiatric Care Dnipropetrovsk Regional Council”, Dnipro, Ukraine

## Abstract

**Introduction:**

Psychobiotics are a group of probiotics that affect the central nervous system related functions and behaviors mediated by the gut-brain-axis via immune, humoral, neural, and metabolic pathways to improve not only the gastrointestinal function but also the antidepressant and anxiolytic capacity.

**Objectives:**

To assess the efficacy the combination of selective serotonin reuptake inhibitors antidepressants (SSRI) and probiotic supplement containing Lactobacillus Plantarum CECT7485 and Lactobacillus Brevis CECT7480 (PLANTARUM) in patients with mild-to-moderate depression.

**Methods:**

Sixty patients with mild-to-moderate depression (according to ICD-10 diagnostic criteria for mixed anxiety and depression disorder, F41.2) were included in an 8-week open label study. Thirty participants received either SSRI antidepressants with PLANTARUM at a dose of 1.0 × 10^9^ CFU once per day. Thirty patients received SSRI antidepressants only without probiotics intake. The severity of depressive symptoms was assessed using Hamilton Depressive Rating Scale (HDRS) and Patient Health Questionnaire (PHQ-9).

**Results:**

After 8 weeks intervention, a clinically significant reduction of HDRS total score (from 45,6±6,1 to 22,5±3,7) was detected in patients with mild-to-moderate depression who received SSRI antidepressants and PLANTARUM (p˂0,001), compared with participants who didn’t receive probiotics (p>0,05). A significant reduction of PHQ-9 total score (from 19,3±2,9 to 9,0±1,9) was identified in patients with mild-to-moderate depression who received SSRI antidepressants and PLANTARUM (p˂0,05). However, the participants received SSRI antidepressant only didn’t meet a clinically significant reduction depressive symptoms (p>0,05) by PHQ-9 scale.

**Conclusions:**

The combination use of SSRI antidepressants and probiotic supplement PLANTARUM significantly reduced the depressive symptoms.

**Disclosure of Interest:**

None Declared

